# *Vibrio cholerae* O1 Imported from Iraq to Kuwait, 2015

**DOI:** 10.3201/eid2209.160811

**Published:** 2016-09

**Authors:** Asish Kumar Mukhopadhyay, Khalifa Al Benwan, Prosenjit Samanta, Goutam Chowdhury, M. John Albert

**Affiliations:** National Institute of Cholera and Enteric Diseases, Kolkata, India (A.K. Mukhopadhyay, P. Samanta, G. Chowdhury);; Al Amiri Hospital, Sharq, Kuwait (K. Al Benwan);; Kuwait University Faculty of Medicine, Jabriya, Kuwait (M.J. Albert)

**Keywords:** Vibrio cholerae O1, Iraq, Kuwait, bacteria, atypical strain, cholera, enteric infections

**To the Editor:** The etiologic agent of the sixth pandemic of cholera was classical biotype of *Vibrio cholerae* O1. The ongoing seventh pandemic is caused by El Tor biotype. The biotypes are differentiated by phenotypic and genotypic characteristics. However, this differentiation blurred when *V. cholerae* O1 strains were detected in Matlab, Bangladesh, in 2006, in which characteristics were mixed. Genetically, the differences occurred in *tcpA*, which encodes the major adherence antigen *rstR* that regulates site-specific recombination of CTXϕ phage and *ctxB* that encodes the B subunit of cholera toxin. These genes had the characteristics of classical biotype in Matlab variants of El Tor strains. Later, various types of El Tor variants were reported in Southeast Asia, Africa, and Haiti. Differentiating features also occur in repeat toxin A gene (*rtxA*), chromosomal location of CTXϕ, the number of heptad repeats in *ToxR* binding region, and the occurrence of vibrio seventh pandemic islands I and II (*1*,*2*).

Kuwait is free of endemic cholera, but imported cases occur there (*3*). Cholera is endemic to neighboring Iraq. An outbreak caused by *V. cholerae* O1 Inaba serotype started in Iraq in September 2015 (*4*). However, a full characterization of the strain is lacking. A thorough characterization of the strain assumes urgency in light of the spread of variants. We characterized isolates from 2 recent cholera cases imported to Kuwait from Iraq.

The first case was in a 19-year-old Kuwaiti man who visited Najaf and Karbala in Iraq in September 2015; the second case was in a 52-year-old Kuwaiti woman who visited the same 2 locations in October 2015. Both had watery diarrhea 3–4 times daily and vomiting; they returned to Kuwait and were admitted to Al Amiri Hospital (Sharq, Kuwait). They gave histories of drinking local water in Iraq, had moderate dehydration, and were treated with intravenous rehydration solution and a single doxycycline dose (500 mg). Diarrhea resolved after 2–3 days. 

Fecal specimens collected at admission from both patients grew yellow colonies on thiosulfate bile salt sucrose agar (Eiken, Tokyo, Japan); these colonies were confirmed as *V. cholerae* O1 Inaba serotype by biochemical reactions and agglutination with specific antiserum (Denka Seiken, Tokyo, Japan). The woman’s isolate was designated as Kuwait 36 and the man’s as Kuwait 37. The isolates were positive for chicken cell agglutination and Voges-Proskauer tests and were polymyxin B resistant, characteristics of El Tor biotype. The isolates were resistant to nalidixic acid but susceptible to ciprofloxacin, norfloxacin, ofloxacin, tetracycline, meropenem, ampicillin, ceftriaxone, trimethoprim/sulfamethoxazole, chloramphenicol, erythromycin, azithromycin, streptomycin, neomycin, and gentamicin by disk diffusion test. Tetracycline susceptibility confirmed favorable response to doxycycline.

We studied the genotype of *ctxB* using a double-mismatch amplification mutation PCR (i.e., mismatches in both primers). PCR with classical *ctxB*-specific primers ctxBF4/ctxBRvCla yielded an amplicon of 191 bp, but not with Haitian *ctxB* specific primers ctxBF3/ctxBRvCla, indicating that the isolates had a *ctxB* of classical biotype (genotype 1) (*5*,*6*). Mismatch amplification mutation assay PCR (MAMA-PCR, i.e., mismatch in only 1 primer) with Haitian-specific *tcpA* primers tcpAF2/tcpARev produced an amplicon of 167 bp but not with El Tor *tcpA*–specific primers tcpAF1/tcpAElRev, suggesting these isolates had the Haitian variant *tcpA* (*2*). MAMA-PCR for *rtxA* with El Tor–specific primer pair rtxAF/rtxAR1 yielded a 187-bp amplicon but no amplicon for Haitian variant primer pair rtxAF/rtxAR2, suggesting the occurrence of *rtxA* of El Tor variety (*2*). The isolates possessed El Tor type *rstR* because they produced a 500-bp amplicon with primer pair rstR2/rstA3R (*7*). The isolates were positive for *rstC*, a repeat sequence activator found in El Tor biotype, because they yielded an amplicon of 238 bp with primer pair rstC1/rstC2 (*8*). *rstB* is required for CTXϕ phage integration. The Haitian strain has a GTA deletion at positions 77–79. MAMA-PCR with primer pair rstB F1/rstB R1 produced a 160-bp amplicon, suggesting the absence of deletion in El Tor type *rstB* (*2*). The isolates had CTXϕ integrated in the large chromosome with RS element downstream because they produced a 766-bp amplicon with CII F/CII R primers (*9*). PCR sequencing with primers Zot F/ctxA R indicated the presence of 4 heptad (TTTTGAT) repeats in the *ToxR* binding region of *ctxAB* promoter, similar to El Tor biotype (*2*). Both isolates possessed vibrio seventh pandemic islands I and II, typical of El Tor biotype as assessed by PCRs with a variety of primers (*10*). Clonal relationship studied by pulsed-field gel electrophoresis suggested that isolates from the Kuwaiti patients were similar to each other and closer to Indian isolates of 2004 (Figure). Cholera is endemic to India; many El Tor variants circulate there (*2*).

**Figure:**
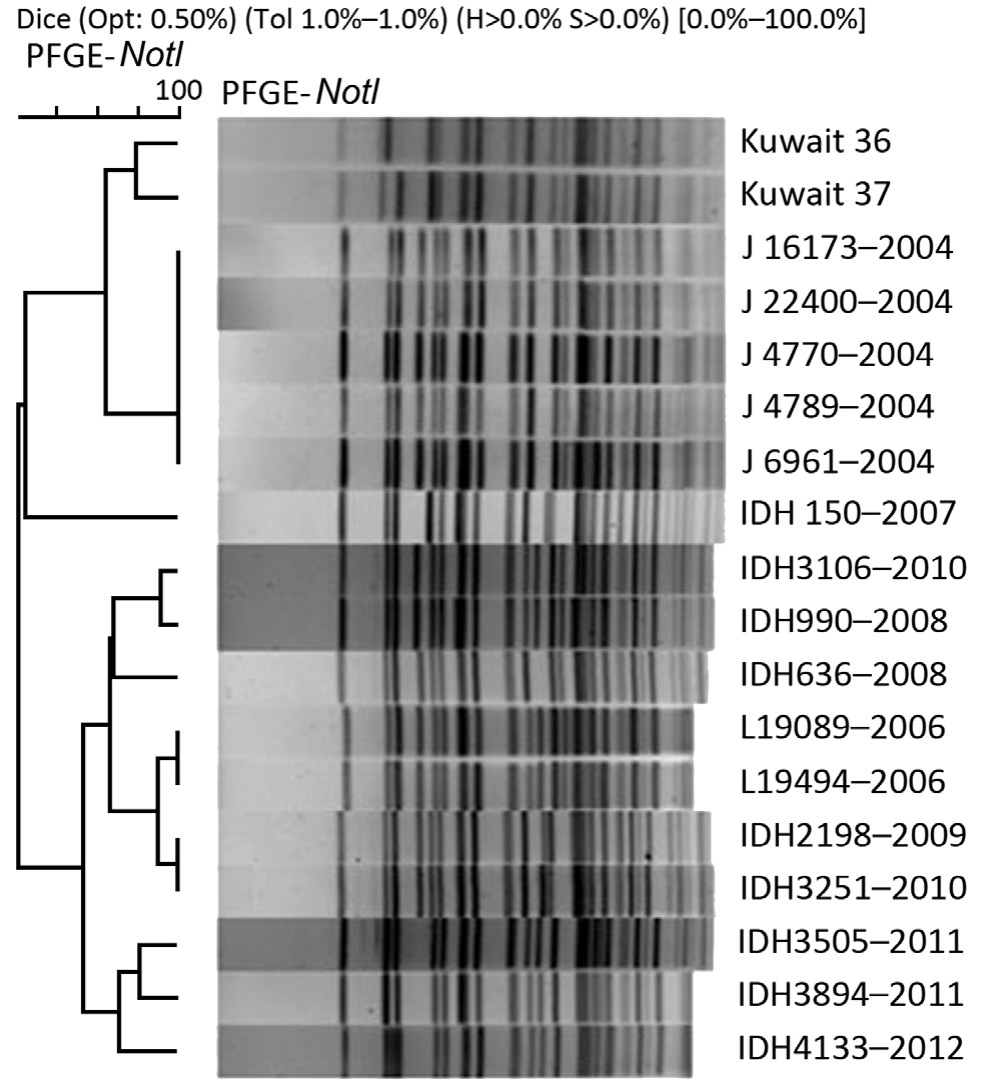
Comparison of PFGE patterns of *Not*I-digested chromosomes of *Vibrio cholerae* O1 isolates from Kuwait with those of isolates obtained from various years (indicated by last 4 digits) from Kolkata, India. The digested chromosomes were separated on CHEF MAPPER (Bio-Rad, Hercules, CA, USA) and dendrogram constructed and analyzed by Bionumerics software (Applied Maths, Sint-Martens-Latem, Belgium). PFGE, pulsed-field gel electrophoresis.

We showed that the strain causing cholera in Iraq did not have the typical El Tor characteristics but instead had mixed characteristics of El Tor, classical, and Haitian strains. Altered strains of *V. cholerae* O1 might have implications for disease severity and vaccine efficacy (*1*). El Tor variants seem to be sweeping the world. We wonder whether they could replace the archetypal El Tor strain and become the causative agent of the eighth pandemic of cholera.
